# Structural basis of antiviral activity of peptides from MPER of FIV gp36

**DOI:** 10.1371/journal.pone.0204042

**Published:** 2018-09-21

**Authors:** Manuela Grimaldi, Ilaria Stillitano, Giuseppina Amodio, Angelo Santoro, Michela Buonocore, Ornella Moltedo, Paolo Remondelli, Anna Maria D’Ursi

**Affiliations:** 1 Department of Pharmacy, University of Salerno, Fisciano (SA), Italy; 2 Department of Medicine and Surgery, University of Salerno, Baronissi (SA), Italy; Università di Napoli Federico II, ITALY

## Abstract

Feline immunodeficiency virus (FIV) is a naturally occurring *Lentivirus* causing acquired immunodeficiency syndrome in felines. It is considered a useful non-primate model to study HIV infection, and to test anti-HIV vaccine. Similarly to HIV, FIV enters cells via a mechanism involving a surface glycoprotein named gp36. C8 is a short synthetic peptide corresponding to the residues ^770^WEDWVGWI^777^ of gp36 membrane proximal external region (MPER). It elicits antiviral activity by inhibiting the fusion of the FIV and host cell membrane. C8 is endowed with evident membrane binding property, inducing alteration of the phospholipid bilayer and membrane fusion. The presence and the position of tryptophan residues in C8 are important for antiviral activity: the C8 derivative C6a, obtained by truncating the N-terminal ^770^WE^771^ residues, exhibits conserved antiviral activity, while the C8 derivative C6b, derived from the truncation of the C-terminal ^776^WI^777^, is nearly inactive. To elucidate the structural factors that induce the different activity profiles of C6a and C6b, in spite of their similarity, we investigated the structural behaviour of the two peptides in membrane mimicking environments. Conformational data on the short peptides C6a and C6b, matched to those of their parent peptide C8, allow describing a pharmacophore model of antiviral fusion inhibitors. This includes the essential structural motifs to design new simplified molecules overcoming the pharmacokinetic and high cost limitations affecting the antiviral entry inhibitors that currently are in therapy.

## Introduction

37 million people are living with human immunodeficiency virus (HIV) infection (www.unaids.org). The so-called “highly active antiretroviral therapy” (HAART), [1–3] combinatorial use of 3 or 4 antiretroviral therapeutics (ARTs) determined reduction of the viral load and extension of the patients’ lives. [4, 5] However, for an increasing number of patients these medicines lost their efficacy due to the emergence of resistant HIV variants. Development of alternative drugs exhibiting broad and sustained antiretroviral activity against HIV-1 remains a pursued goal to date. [6]

In view of the development of new anti-HIV therapy targeting the single steps of HIV life cycle, thousands of molecules have been tested. Among these, many have been designed and screened as virus entry inhibitors. [6, 7] Nowadays, enfuvirtide is the only effective entry inhibitor approved for use as anti-HIV in humans. [8–10] It is used in combined therapy with other anti-HIV drugs, but owing to its peptide nature—responsible for poor pharmacokinetic and high economic cost-, it has a restricted clinical application.

A crucial event for the entry of HIV in host cell membranes is the conformational rearrangement of the envelope glycoprotein gp41. During the structural refolding of gp41, more than one conformational events synergically work to realise the fusion of virus and cell membranes: i) the C-terminal heptad repeats (CHR or HR2) come in close proximity with the N-terminal heptad repeats (NHR or HR1); ii) the extreme hydrophobic “membrane proximal external region” (MPER) is exposed to the host cell and interacts with lipid surface, destabilizing the lipid bilayer. [11–14] Many molecules have been screened as potential inhibitors of CHR-NHR interaction in gp41 [15–19]; others as potential inhibitors of the binding between MPER and the host cell membrane. [20]

Among the molecules tested for their ability to prevent the interaction of MPER with the host cell membrane, we have extensively studied C8, an octapeptide corresponding to the sequence 770–777 of MPER in gp36 glycoprotein of feline immunodeficiency virus (FIV). [21]

FIV is the pathogen of the acquired immunodeficiency syndrome (AIDS) in felines. [22–24] It exhibits biological properties very similar to HIV, and as such it is considered a useful non-primate model to study HIV infection, and to test anti-HIV vaccine [25–27] and drugs. Analogously to HIV, FIV [28] enters cells thanks to gp36, the envelope glycoprotein [29–31] showing the same structural and functional properties of gp41. [32–34] C8, including three equally spaced Trp residues in its sequence, elicits antiviral activity preventing the entry of the virus in the host cells. C8 is endowed with strong membrane binding property, and many evidence show that its antiviral action is based on its ability to prevent the interaction of gp36 MPER with the surface of the host cell. [35]

Previously, we extensively investigated the membrane active properties of C8 using different physical-chemical methodologies. [36] C8 was studied by circular dichroism (CD) and nuclear magnetic resonance (NMR) spectroscopy, showing β-turn conformations in micelle solution; the regularity of this structure varies with the charge of the micelle surface and the positioning of the Trp side chains on the lipid surface. [36–39] C8 was analysed by EPR and fluorescence spectroscopy and the results were validated by molecular dynamic calculations: in lipid vesicles of varying complexity and composition, C8 was able to disrupt phospholipid bilayers, reducing membrane thickness and inducing membrane fusion. [36–39]

**Fig 1:**
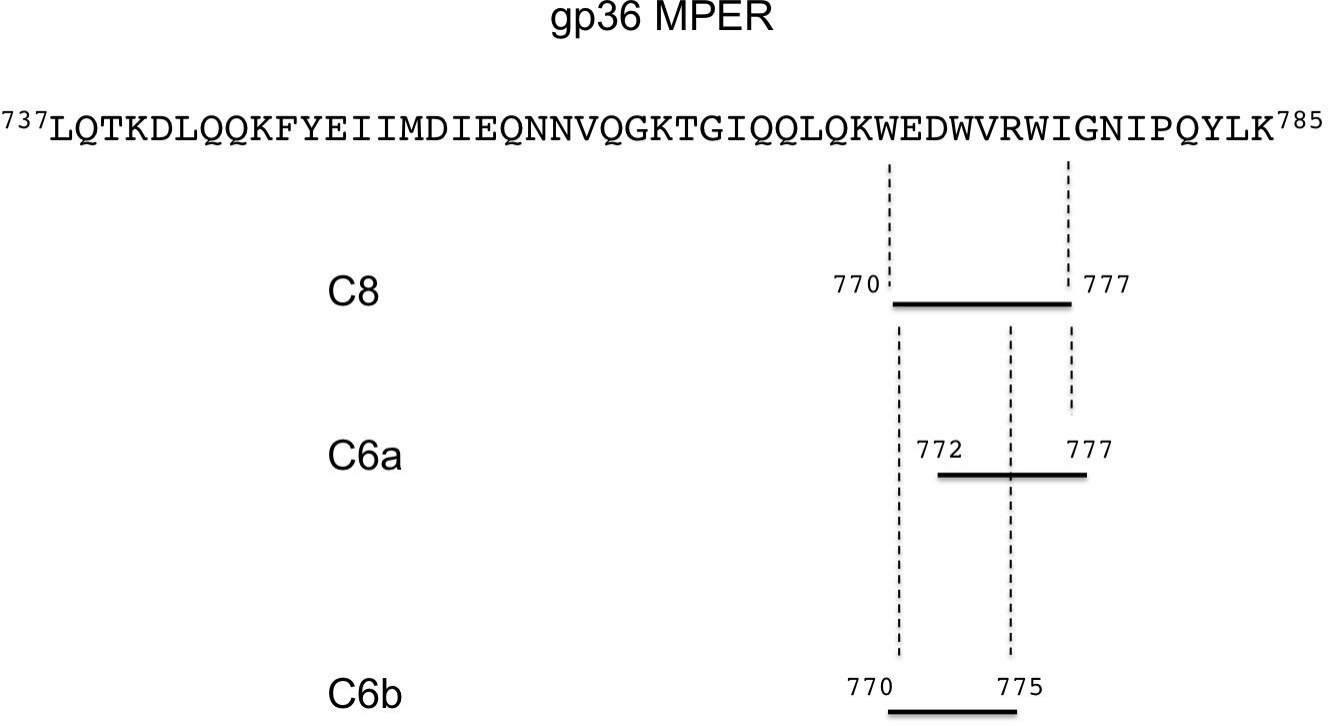
Graphic representation of the gp36 MPER domain of FIV coat glycoprotein. The fragments relative to C8, C6a and C6b peptide sequences are highlighted.

Structure-activity relationship analysis on shorter synthetic derivatives of C8 revealed the importance of Trp residues for the antiviral activity. [37] Experiments performed to test the inhibition of the replication of primary FIV isolates in lymphoid cells showed that the fragment of C8 (C6a) deriving from the truncation of the C8 N-terminal residues ^770^WE^771^ (Fig 1), showed a twofold IC_50_ activity compared to C8 (C8: IC_50_ 0.06 and 0.05 μg/ml; C6a: IC_50_ 0.15 and 0.06 μg/ml) on the viral strains FIV-M2 and FIV-Pet, respectively. [35] On the contrary, the fragment of C8 (C6b) deriving from the truncation of the C8 C-terminal residues ^776^WI^777^ was nearly inactive, with IC_50_ values for FIV-M2 and FIV-Pet > 50 μg/ml. [35]

In the present work, we report the conformational analysis of C6a and C6b by CD and NMR spectroscopy in micelle solution composed of dodecylphosphocholine (DPC)/sodium dodecyl sulphate (SDS) 90:10 M/M. On a higher dimension scale, we studied C6a and C6b using confocal microscopy imaging on vesicles characterized by different phospholipid compositions. [40] Our data allow identifying the conformational factors determining the different C6a and C6b membrane binding properties and hence the different antiviral activity profiles.

One major concern relative to the anti-HIV fusion inhibitors is their peptide chemical structure and the high number of residues. [41] A valuable quality of C8 and its derivatives is the small size. The structural features of C6a and C6b derived from our analysis, matched to those of their parent peptide C8, lead to describe a pharmacophore model for anti-HIV fusion inhibitors; this includes the essential structural motifs to design new simplified molecules overcoming the limitations affecting the entry inhibitors that currently are in clinical application.

## Materials and methods

### Peptide synthesis

The C8, C6a and C6b peptides were synthesized by the Fmoc solid phase synthesis method according to the previously published procedure. [36] For the confocal microscopy analysis, all peptides labelled with nitrobenzoxadiazole (NBD) fluorophore and the L-diamino propionic acid Fmoc-Dap(NBD)-OH) [40] were used in substitution of ^770^W for C6b and ^773^W for C6a. Labelled C8 peptide was prepared by synthesizing Ac-^770^Dap(NBD)EDWVGWI^777^-NH_2_, the C6b peptide was prepared by synthesizing Ac-^770^Dap(NBD)EDWVG^775^-NH_2_, and the C6a peptide was prepared by synthesizing Ac-^772^D-Dap(NBD)-VGWI^777^-NH_2_.

### Confocal microscope Imaging of liposomes

The multilamellar lipid vesicles (MLVs) of DOPC, DOPG and DOPC/DOPG (90:10, 70:30, 30:70, and 10:90 M/M) were prepared by mixing appropriate amounts of lipids, dissolved in dichloromethane/methanol mixture (25 mM lipid concentration), in a round-bottom test tube. The total weight of the lipid for each sample was 1.0 mg. MLVs were prepared from lipid DOPC and DOPG solutions that were desiccated, dried overnight and hydrated in a 10 mM phosphate buffer at pH 7.4. MLVs were prepared in six different lipid compositions: DOPC only, DOPG only, DOPC/DOPG 90:10 M/M, DOPC/DOPG 70:30 M/M, DOPC/DOPG 30:70 M/M and DOPC/DOPG 10:90 M/M. MLVs, in the presence of NBD-labelled C8, NBD-labelled C6a and NBD-labelled C6b peptides, were prepared by adding during their formation, the labelled peptides at a concentration of 0.5 mM (lipid to peptide 50:1 M/M). Lipid solutions in the presence or absence of labelled peptides were used for microscopy analysis. [42]

A total of 10 μL of each solution, in the presence or absence of NBD-labelled C8, NBD-labelled C6a and NBD-labelled C6b peptides was taken and spotted onto a cover slip. Images were acquired as previously described [43, 44] on a laser scanning confocal microscope (LSM 510; Carl Zeiss MicroImaging) equipped with a plan Apo 63X, NA 1.4 oil immersion objective lens. For each field, both fluorescent and transmitted light images were acquired on separate photomultipliers and were analysed with Zeiss LSM 510 4.0 SP2 software. In samples in which different z-planes were distinguishable, a z-stack acquisition mode was performed to focus a single z-plane, as published previously. [45]

### CD analysis

CD experiments were performed at 25°C on an 810-Jasco spectropolarimeter as an average of 4 scans with 10 nm/min scan speed, 4 s response time and 2 nm bandwidth, using a quartz cuvette with a path length of 1 mm, a measurement range from 190 to 260 nm (far UV), at temperature of 25°C. Throughout the measurements, the trace of the high-tension voltage was verified to be less than 700 V, which ensures the reliability of the obtained data.

Far UV CD spectrum of only mixed micelles (DPC/SDS 90:10 M/M (27 mM/3 mM)) in aqueous solution (pH 7.4, 10 mM phosphate buffer containing H_2_O) was acquired as reference, than 500 μM of C6a and C6b peptides were added to mixed micelles for the conformational analysis. [46] The processed curves of C6a and C6b peptides in mixed micelles were obtained by using Spectra Analysis tool of Jasco software. The CD curves of C6a and C6b peptides in mixed micelles were corrected for the solvent contribution by subtraction of CD reference spectrum and then final CD spectra were obtained after baseline correction and binomial smoothing. Those resulting spectra were used for the estimation of the secondary structure content using the algorithms CONTIN and SELCON from the DICHROWEB website. [47]

### NMR analysis

Samples for the NMR experiments were prepared at 1 mM C6a and C6b concentrations, in a mixture of *d*_*38*_-DPC or *d*_*25*_-SDS 90:10 M/M (27 mM/3 mM) in aqueous solution (pH 7.4, 10 mM phosphate buffer containing H_2_O/D_2_O). [46] For the spin label experiments, the 5- and 16-doxylstearic acids were solubilized in dimethyl sulfoxide-d_6_ and then added to the samples.

The NMR spectra were recorded on a Bruker DRX-600 spectrometer. All samples had a recording of the 1D ^1^H homonuclear spectra in the Fourier mode, with quadrature detection and two-dimensional (2D) TOCSY and NOESY spectra in the phase–sensitive mode using quadrature detection in *ω*1 by time–proportional phase incrementation of the initial pulse. [48–50] Water signal was suppressed using WATERGATE pulse sequence experiments. [51] Data block sizes contained 2048 addresses in t_2_ and 512 equidistant t_1_ values. Prior to Fourier transformation, the time domain data matrices were multiplied by shifted sin^2^ functions in both dimensions. A mixing time of 70 ms was used for the TOCSY experiments. The NOESY experiments were run at 300 K with mixing times in the range of 100–300 ms. Qualitative and quantitative analyses of TOCSY and NOESY spectra were carried out using SPARKY software. [52]

### NMR structure calculations

Peak volumes were translated into upper distance bounds with the CALIBA routine from the CYANA software package. [53] The requisite pseudo-atom corrections were applied for non-stereo specifically assigned protons at prochiral centres and for the methyl group. After discarding redundant and duplicated constraints, the final list of experimental constraints was used to generate a group of 100 structures by the standard CYANA protocol of simulated annealing in the torsion angle space (using 10.000 steps). No dihedral angle or hydrogen bond restraints were applied. The best 20 structures that had low target function values and small residual violations were refined by *in vacuo* minimization in the AMBER 1991 force field using the SANDER program of the AMBER 5.0 suite. [54, 55] In order to mimic the effect of solvent screening, all net charges were reduced to 20% of their true values. Additionally, a distance-dependent dielectric constant (ε = r) was used. The cut-off for non-bonded interactions was 12 Å. The NMR-derived upper bounds were imposed as semi-parabolic penalty functions, with force constants of 16 Kcal/mol Å^2^. The function was shifted to be linear when the violation exceeded 0.5 Å. The best 10 structures after minimization had AMBER energies ranging from -441.4 to -391.1 Kcal/mol. The final structures were analysed using AutoDock 4.2 software. [56]

## Results

### Confocal microscopy imaging

**Fig 2:**
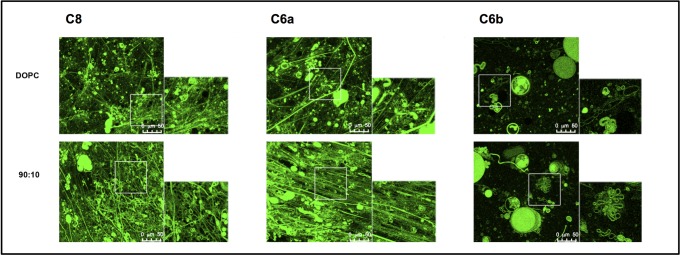
Confocal microscopy images of MLVs in presence of NBD labelled C8, C6a and C6b peptides. MLVs are composed of DOPC/DOPG 90:10 M/M. Images were acquired on a laser scanning confocal microscope (LSM 510; Carl Zeiss MicroImaging) equipped with a plan Apo 63X, NA 1.4 oil immersion objective lens. For each field, both fluorescent and transmitted light images were acquired on separate photomultipliers and were analysed with Zeiss LSM 510 4.0 SP2 software.

**Fig 3:**
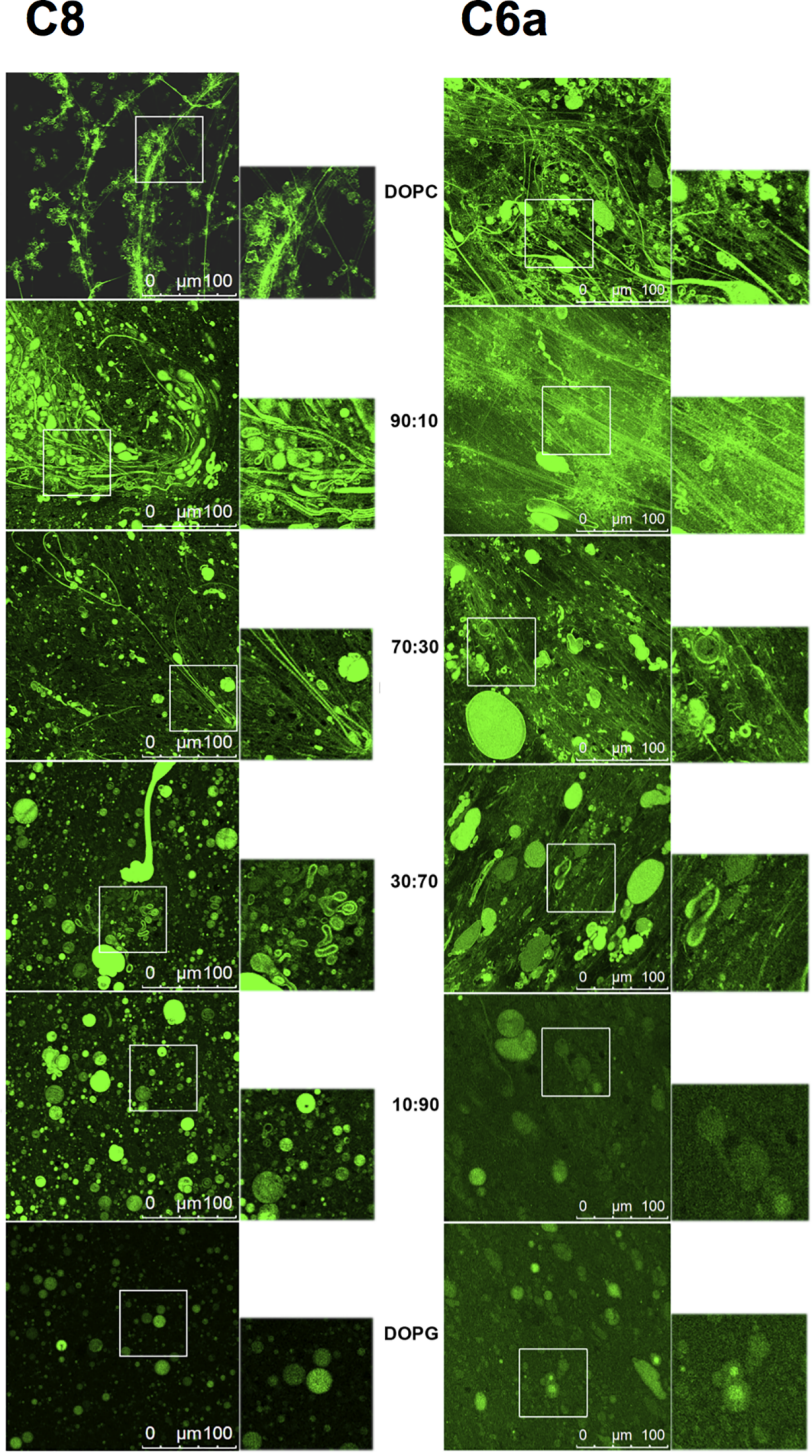
Confocal microscopy images of MLEVs in presence of NBD labelled C8 (left) and C6a (right) peptides. MLEVs vary in DOPC/DOPG composition moving from DOPC/DOPG 100:0 M/M (top) to DOPC/DOPG 0:100 M/M (bottom). Images were acquired on a laser scanning confocal microscope (LSM 510; Carl Zeiss MicroImaging) equipped with a plan Apo 63X, NA 1.4 oil immersion objective lens. For each field, both fluorescent and transmitted light images were acquired on separate photomultipliers and were analysed with Zeiss LSM 510 4.0 SP2 software.

Confocal microscopy imaging of C6a and C6b was carried out in MLVs of different DOPC/DOPG compositions. To evaluate the behaviour of C6a and C6b in MLVs characterized by different membrane charge, we used MLVs composed of zwitterionic DOPC, negatively charged DOPG and DOPC/DOPG varying in composition (90:10; 70:30; 30:70; 10:90 M/M). The peptides were labelled with NBD fluorophore [40] to examine their localization on the vesicles. MLVs were imaged alone and in the presence of the labelled peptides. (Figs 2 and 3) In absence of peptide, MLVs assume spherical shape, with diameter ranging from 5 to 20 μm; addition of C6a (NBD^773^)W and C6b(NBD^770^)W induces fluorescence of the lipid membranes, indicating the localization of the peptides on the lipid surface. Figs 2 and 3 report confocal microscopy images of MLVs composed of DOPC/DOPG 90:10 M/M, in presence of NBD labelled C8, C6a and C6b peptides.

Fig 2 shows that in MLVs composed of DOPC/DOPG 90:10 and including C6b, the diameter ranges between 5 and 10 μM; only sporadically membrane fusion occurs, generating MLVs up to 50 μM diameter. In the presence of the C6a peptide, highly dynamic budding and fusion events are observable: large spherical liposomes characterized by 5–20 μM diameter, often appear because of the impaired budding of the smaller vesicles. Notably, MLVs are interconnected through the formation of straight-chain tubular structures (Fig 3: DOPC, 90:10, 70:30 panels) that often extend throughout the entire field of view and beyond and are typically capped at both ends with large liposomal structures. These “tubulation” events occur in different z-planes, forming a crowded three-dimensional network of tubules and liposomes.

The frequency of tubule formation is high in DOPC and 90:10 DOPC/DOPG samples and decreases with the reduction of DOPC concentration, until nearly disappearing in the 10:90 DOPC/DOPG and fully DOPG samples. A similar pattern of tubulation and budding is shown by MLVs in the presence of C8 peptide (Figs 2 and 3), indicating that the residues ^772^D-I^777^, corresponding to the C6a sequence, have the structural requirements necessary for the membrane active property.

### CD and NMR conformational analysis

The conformational behaviour of C6a and C6b was studied in mixed DPC/SDS (90:10 M/M) micelles using CD and NMR spectroscopy. [46] Micelle solutions are often used as a biomimetic membrane model to study the structural features of membranotropic molecules. They are made of surfactants, such as the zwitterionic DPC and the negatively charged SDS, at concentrations much higher than their critical micelle concentration (c.m.c.). These surfactants form spherical aggregates where the polar head groups are located on the surface and the hydrophobic tails point to the centre. Micelle solutions are ideal systems for solution CD and NMR spectroscopy, as they tumble sufficiently quickly to result in high-resolution spectral lines. [57, 58]

**Fig 4:**
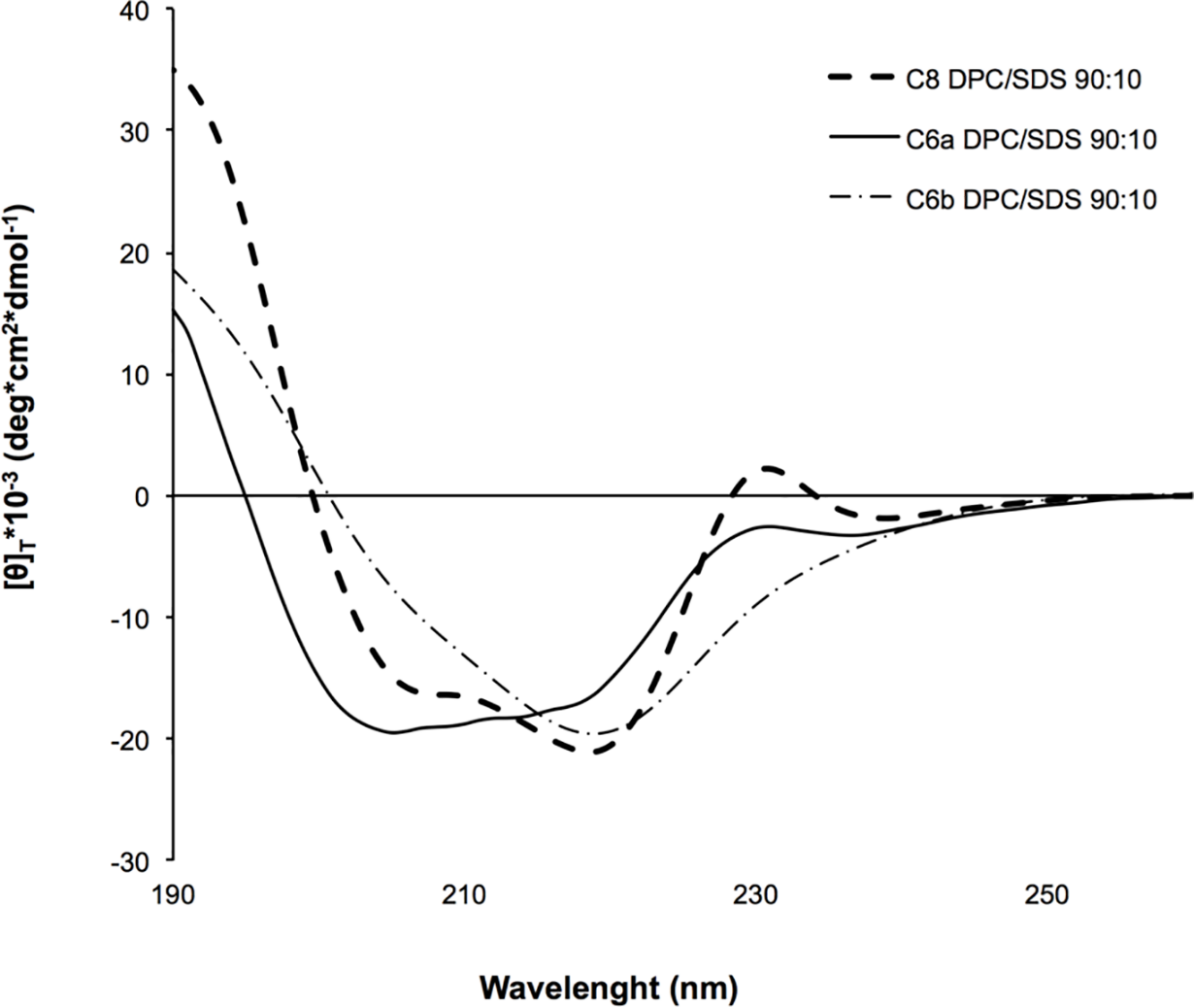
Processed far UV CD spectra of C8, C6a and C6b peptides in DPC/SDS 90:10 M/M. The CD spectra were acquired using a JASCO 810 spectropolarimeter at room temperature with a cell path length of 1 mm. The measurement range spans from 190 to 260 nm.

Fig 4 shows the far UV CD spectra of C6a and C6b recorded in DPC/SDS 90:10 M/M micelle solution. The shapes of the far UV CD spectra are consistent with the prevalence in C6a of regular turn-helix conformation, and non-canonical secondary structures in C6b. Quantitative evaluation of the CD spectra using CONTINN algorithm (DICHROWEB website) [47] confirm these indications.

**Fig 5:**
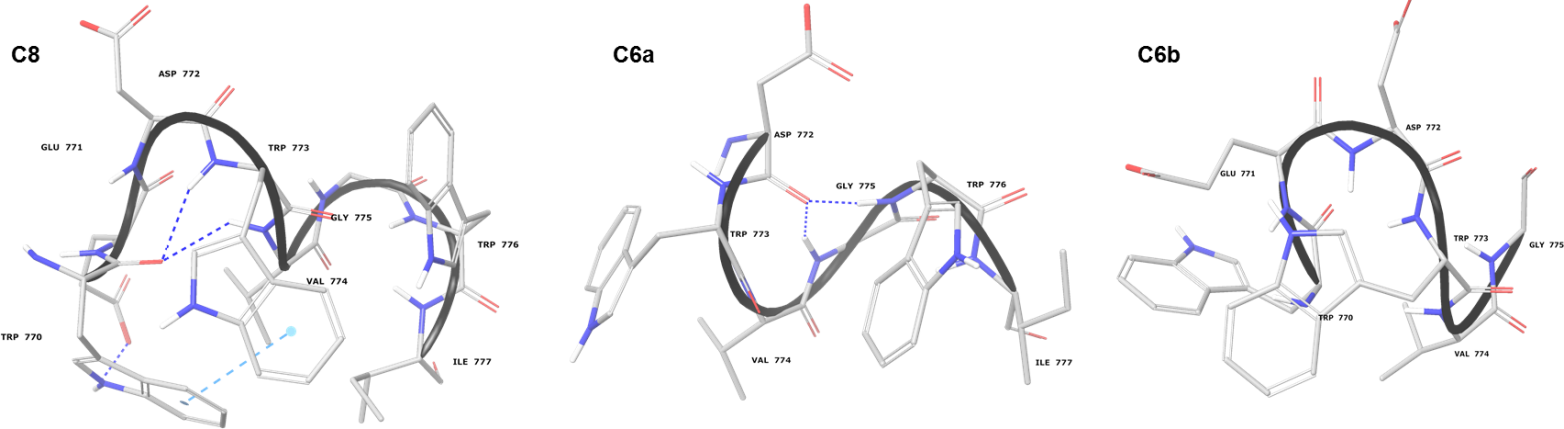
Low energy NMR models calculated for C8, C6a and C6b peptides in DPC/SDS 90:10 molar ratio. The calculation was carried out using standard CYANA protocol of simulated annealing in torsion angle space.

NMR spectra of C6a and C6b in the same mixed DPC/SDS micelle solution (90:10 M/M) were acquired using a Bruker DRX-600 spectrometer. 2D COSY, TOCSY and NOESY data were recorded to allow for chemical shift assignment, according to the conventional procedure of Wuthrich. [59] The proton chemical shifts are reported in the Supporting Information (S1 and S2 Tables). COSY, TOCSY and NOESY spectra were analysed using SPARKY software. [52] The sequential and medium-range connectivities observed in the NOESY spectra (S2 Fig) indicate the presence of folded structures in both the peptides. NOE data were transformed in interprotonic distances for the calculation of C6a and C6b NMR structures using CYANA software. [60] Fig 5 shows the NMR structure bundles of C6a and C6b. 20 structures out of 50 calculated were selected according to the lowest values of the target function. [60] The selected conformers were superimposed at level of the backbone heavy atoms showing 0.32 Å and 0.47 Å RMSD for C6a and C6b respectively. Analysis of the backbone dihedral angles by using PROMOTIF [61] indicates the prevalence in C6a of regular β-turn structures encompassing the residues ^773^W-W^776^ and stabilized by H-bonds between the carbonyl of ^772^D and the HN of ^775^G and ^776^W (22 out of 50 calculated structures) (Fig 5). Inspection of the side chains in C6a NMR structures evidences a parallel orientation of the Trp indolyl rings, to expose a large hydrophobic surface.

Analysis of C6b NMR structure bundle indicates the prevalence of non-canonical secondary structures. The amino acid side chains form a globular amphipathic structure exposing, on one side, the negatively charged side chains of ^771^E and ^772^D, and on the other side the hydrophobic indolyl rings of ^770^W and ^773^W.

An estimation of the molecular surface, calculated according to the aptitude of the peptides to engage electrostatic/polar interaction with positively/negatively charged surfaces, or hydrophobic interactions with hydrophobic surfaces (Supplementary information, S3 Table) [56] indicates that C6a and C6b expose 286.2 Å^2^ and 220.5 Å^2^ hydrophobic surface respectively. A similar calculation for the NMR structure of the parent peptide C8, indicate that the extension of the hydrophobic surface of C8 is 288.3 Å^2^, very similar to that of C6a. Moreover, the molecular surfaces of C6a and C6b and of their parent peptide C8 shows several spots of electrostatically charged surfaces. According to molecular dynamic calculations performed by us, [38] these charged sites engage electrostatic interactions with the polar heads of the phospholipids. Hence, hydrophobic and electrostatic interactions are synergic to destabilize the phospholipid bilayer and modify membrane size and shape.

### Spin-label studies

The NMR analysis in DPC/SDS 90:10 M/M micelle solutions was the starting point to investigate the positioning of the peptides with respect to the surface and interior of the micelle using 5-doxylstearic acid (5-DSA) and 16-doxylstearic acid (16-DSA) as paramagnetic probes. Both of these compounds contain a doxyl head group and a cyclic nitroxide with unpaired electrons that is bound to the aliphatic carbon chain at either position 5 or 16. NMR experiments were recorded in micelle solutions containing 5-DSA or 16-DSA. Paramagnetic probes are able to induce a broadening of the NMR signal and a decrease in the resonance intensities. Generally, the sites of the peptide that are closest to the NO· moiety are affected by the unpaired electrons, with an increase in nuclear relaxation rates and, thus, a decrease in proton signals. If the peptide is close to the surface, a decrease in intensity is observed in the 5-DSA spin-labelled micelles, but if the peptide penetrates the inner core of the micelle, a decrease in intensity is observed in the 16-DSA spin-labelled micelles. [46] 1D and 2D TOCSY spectra of the C6a and C6b peptides were recorded in DPC/SDS 90:10 M/M micelle solutions in the presence and absence of 5-DSA and 16-DSA while keeping all other conditions constant. A comparison of the 1D ^1^H NMR proton spectra of the C6a and C6b peptides recorded in the presence and absence of 5-DSA show a selective perturbation of Trp CHα-NH and Trp-indolyl proton signals in the presence of 5-DSA. Comparison of TOCSY spectra of C6a and C6b recorded in DPC/SDS 90:10 molar ratio micelle solutions in the presence of 5-DSA shows that the Trp Hε1 signals are decreased in intensities due to the presence of the spin label. This effect is nearly 60% in C6a and 30% in C6b. Backbone NHs of Trp are 30% and 15% decreased in intensities for C6a and C6b respectively. This effect is consistent with the localization of the peptide on the surface of micelles, as observed in the confocal microscopy imaging (Figs 2 and 3).

## Discussion

C8 is a tryptophan-rich octa-peptide belonging to the MPER of gp36. [21, 35, 39] It exerts antiviral activity by inhibiting the fusion of the virus with the cell membrane. In membrane models of different complexity and composition, C8 destabilizes phospholipid bilayer and induces membrane fusion. [36–38] C8 includes three equally spaced Trp residues and several evidence suggest that its antiviral molecular mechanism consists in the inhibition of the interaction of gp36 MPER with the surface of the host cell. [35, 36] SAR studies on C8 derivatives indicated that the presence and the position of Trp residues are essential for the antiviral activity: C6a and C6b, resulting from the truncation of the N-terminus ^770^WE^771^ and the C-terminus ^776^WI^777^ respectively, differ in their activity profiles: C6a inhibits the replication of primary FIV isolates in lymphoid cells, whereas C6b is nearly inactive. C6a and C6b are structurally similar: i) both are fragments of C8; as shown in Fig 1, they are characterized by the same amino acid composition, except for one residue; ii) both include two out of the three Trp residues. To elucidate the structural factors causing the different biological activities of C6a and C6b, we performed CD and NMR conformational analysis in mixed micelle solutions composed of zwitterionic DPC and negatively charged SDS. On a larger scale, we analysed by confocal microscopy the effect of the two peptides on MLVs containing zwitterionic DOPC and negatively charged DOPG phospholipids. Confocal microscopy data confirmed the relationship between antiviral activity and the membrane active properties. The interaction with the vesicle surface of the antiviral active peptides C6a and C8, induce perturbation of the bilayer architecture and consequent modification of vesicle size and shape. On the contrary, DOPC/DOPG vesicles remain unmodified in their size and shape upon the action of C6b. Fig 3 shows a comparison of confocal microscopy images of MLVs with different DOPC/DOPG compositions, including either C6a or C8. Both, C6a and C8 induce membrane tubulation in a manner dependent on the DOPC/DOPG ratio. In MLVs characterized by zwitterionic phospholipid content less than 50%, the effect of C6a is an increase of vesicle size possibly consequent to vesicle fusion. In contrast, in MLVs having a zwitterionic phospholipid content greater than 50% (or in pure DOPC vesicles), C6a induces membrane deformation with the appearance of a complex network of membrane tubes. Interestingly, our data indicate that the effect of the peptide on the membrane is critically dependent on the properties of the phospholipid bilayer: this aspect is important and requires attention when considering the risk for virus resistance.

**Fig 6:**
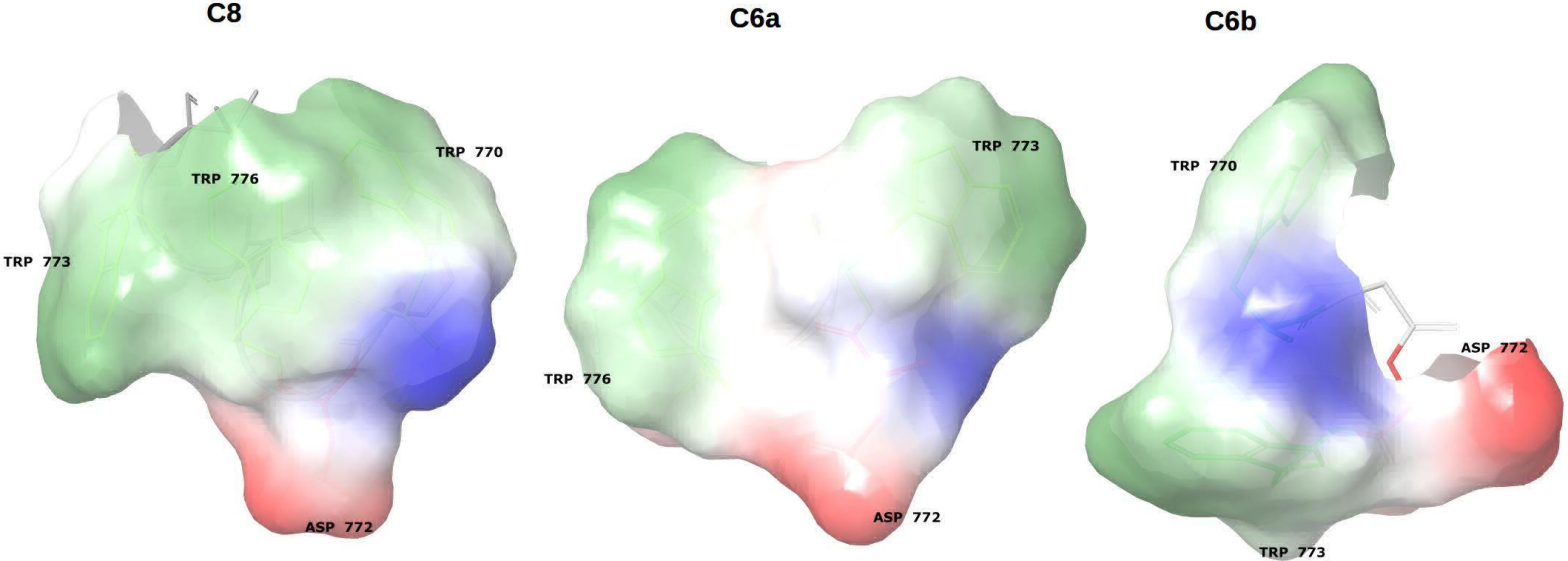
Molecular surface calculated as propensities of the different portions of the molecules to interact according to the specific surface properties. Molecular surfaces of Trp residues are coloured in green-white: they account for the propensity of the peptides to engage hydrophobic interactions. Molecular surfaces of Asp residues are coloured in blue: they account for the propensity of the peptides to engage polar/electrostatic interactions.

As previously reported, the conformations of C6a and C6b were studied in mixed zwitterionic DPC/SDS (90:10 M/M) micelles. Consistent with the observation of a high number of NOE effects in 2D NOESY spectra, the two peptides assume non-*random coil* but well-structured conformation in the micelle solution. This may be explained considering that they are confined to the surface of micelles and have limited conformational freedom. Analysis of the backbone dihedral angles reveals that C6a adopts regular *β-turn* structures involving the residues ^773^WVGW^776^ (S4 Table), while C6b assumes folded, but non-canonical, secondary structures. According to the amino acid sequences, we can infer that in C6a, the ^774^VG^775^ residues act as a flexible linker between ^773^W and ^776^W, favouring a regular *β-turn* conformation. In C6b, ^771^ED^772^ forms a more rigid linker between ^770^W and ^773^W, preventing the formation of regular secondary structures. Analysis of the most representative C6a and C6b NMR structures evidence that C6a is characterised by an elongated molecular shape, with the indolyl rings of ^773^W and ^776^W in a nearly parallel orientation (the planes that run through the ^773^W and ^776^W are at an angle of angle of 13.64°). These generate a hydrophobic molecular surface (Fig 6), calculated for 220.5 Å^2^. The negatively charged ^772^D side chain flanks the ^773^W indolyl ring directly in this molecular area. Contrarily to C6a, C6b has a globular shape, and the planes that run through the ^770^W and ^773^W indolyl rings are at an angle of 62°. The 220.5 Å^2^ molecular surface generated by these residues is smaller as compared to C6a. The negatively charged side chains of ^771^E and ^772^D are on the opposite side with respect to the tryptophan indolyl rings (Fig 6).

**Fig 7:**
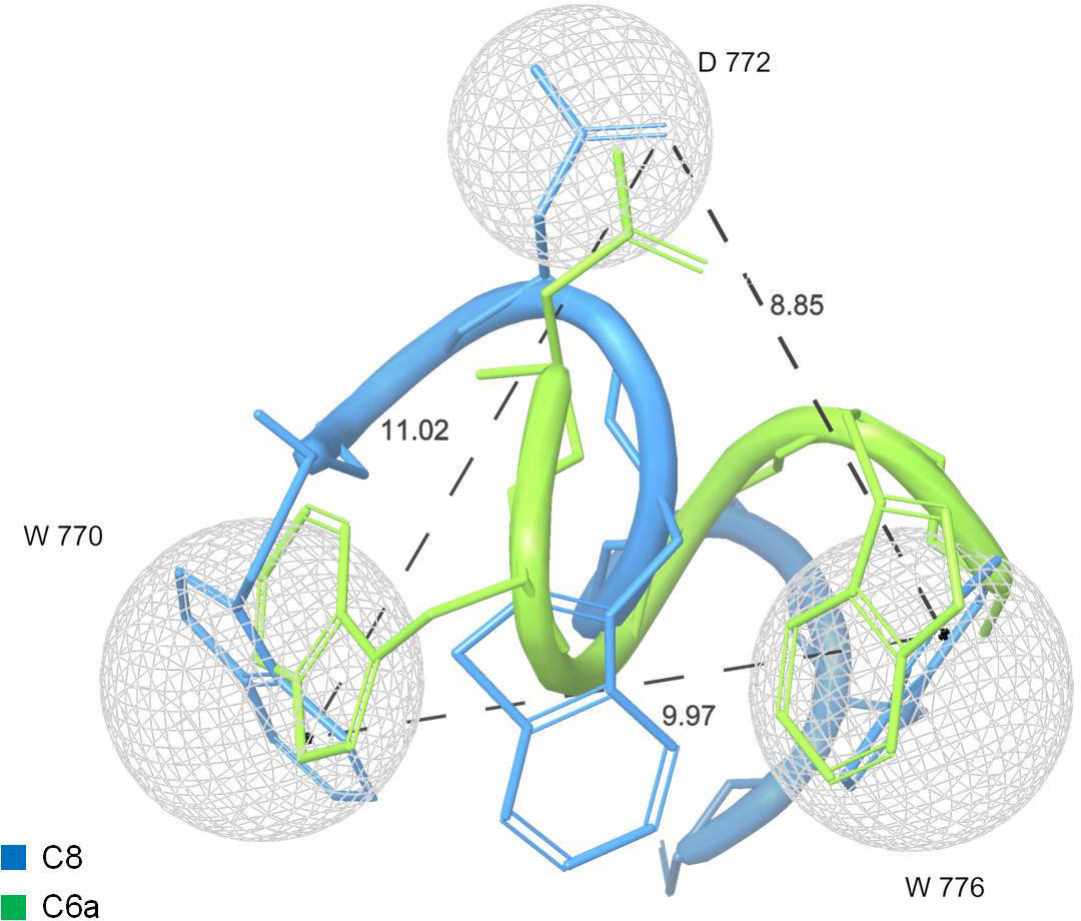
Pharmacophore model of anti-FIV fusion inhibitors. Overlapping of C8 (light blue) and C6a NMR representative models. Molecular surfaces (grey) surround the carboxylic moiety of ^772^D side chain and indolyl rings of ^770^W and ^776^W in C8, and ^773^W and ^776^W in C6a.

Many peptide molecules have been studied as antiviral fusion inhibitors. However, those approved for antiviral therapy have high molecular weight, with complications deriving from the high economic cost and the difficulty of administration. C8 and its derivatives are small peptides. The comparison of the most representative conformations of C8 and C6a (Fig 7) indicates that it is possible to draw a pharmacophore model including the indolyl rings of the two Trp residues (which in C8 belong to ^770^W and ^776^W, and in C6a belong to ^773^W and ^776^W) and the side chain of ^772^D. This model may be the starting point to design new small peptide-peptidomimetic molecules endowed with improved pharmacodynamics/ pharmacokinetic properties.

Using atomistic molecular simulations, [38] we previously demonstrated that the binding of C8- with phospholipid bilayers is mediated by i) hydrophobic interactions involving the Trp indolyl rings and the hydrophobic chains of phospholipids ii) salt bridge interactions established between the negatively charged amino acids (i.e., ^771^E and ^772^D) and the phospholipid zwitterionic polar heads. The pharmacophore shown in Fig 7 is consistent with this model of interaction for C6a and C8, but not for C6b; notably, the absence of the ^770^WE^771^ N-terminal residues may explain the 2 times less activity of C6a as compared to the C8 for FIV-M2. Accordingly, we can speculate that activity at vesicle surface and at cell membrane rely in the synergic molecular interactions involving Trp indolyl rings and negatively charged aminoacid: these induce destabilization of the bilayer, determining membrane tubes and membrane fusion.

## Conclusions

C6a and C6b two derivatives of the C8 anti-FIV peptide, although very similar to their precursor show different activity profile: in particular, C6a preserves anti-viral activity, while C6b is nearly inactive. We analysed C6a and C6b in bio-membrane mimicking environments using confocal microscopy and CD and NMR spectroscopy. Our data provide additional evidence that common antiviral activity profiles correspond to similar membrane binding properties: actually C6a, similarly to C8, has the ability to destabilize membrane vesicles, producing complex network of membrane tubes. Conformational data show that the action of C6a and C8 at membrane level is mediated by interactions involving ^772^D and two Trp residues, which correspond to ^770^W and ^776^W in C8, and ^773^W and ^776^W in C6a. These residues define a pharmacophoric model that can be considered the basis to design simplified molecules, overcoming the pharmacokinetic and high cost limitations affecting the entry inhibitors currently used as anti-HIV therapeutics.

## Supporting information

S1 Table ^1^H chemical shift of C6a. ^1^H chemical shift of C6a in DPC/SDS micelle solution 90:10 M/M.
(DOC)

S2 Table ^1^H chemical shift of C6b. ^1^H chemical shift of C6b in DPC/SDS micelle solution 90:10 M/M.
(DOCX)

S3 Table Molecular surface values. Molecular surface values calculated as propensities to engage molecular interaction according to specific surface properties.
(DOCX)

S4 Table Backbone dihedral angles of C6a and C6b. Backbone dihedral angles of C6a and C6b in DPC/SDS 90:10 M/M micelle solution.
(DOCX)

S1 Fig Sequential and medium range connectivities of C6a and C6b. Sequential and medium range connectivities collected in NOESY spectra of C6a and C6b in DPC/SDS 90:10 M/M.
(DOCX)

S2 Fig **Low field region of the 600 MHz 1D proton spectra of C6a (a) and C6b (b) peptides in presence of spin labels.** Low field region of the 600 MHz 1D proton spectra of C6a (a) and C6b (b) peptides recorded in DPC/SDS 90:10 M/M micelles solutions at 300 K in absence (blue) and in presence of 5-DSA and 16-DSA (red) at a concentration of one spin label *per* micelle.
(DOCX)
