# Supplementary material for: Structural basis of antiviral activity of peptides from MPER of FIV gp36
Source: PLoS One. 2018 Sep 21;13(9):e0204042. doi: 10.1371/journal.pone.0204042 (PMC6150481; doi:10.1371/journal.pone.0204042)
Supplement: S1 Table — 1H chemical shift of C6a in DPC/SDS micelle solution 90:10 M/M. (DOC) [file pone.0204042.s001.doc]

**S1 Table.** **^1^H chemical shift of C6a.** ^1^H chemical shift of C6a in DPC/SDS micelle solution 90:10 M/M.

| **Residue** | **HN** | **CαH** | **CβH** | **CγH** | **CδH** | **CεH** | **Others** |
| --- | --- | --- | --- | --- | --- | --- | --- |
| **Asp^772^** | 8.407 | 4.499 | Qβ 2.723 |  |  |  |  |
| **Trp^773^** | 8.085 | 4.280 | Hβ2 3.305  Hβ3 3.131 |  | Hδ1 7.005 | Hε1 10.541  Hε3 7.380 | HH2 6.947  Hζ2 7.247  Hζ3 6.854 |
| **Val^774^** | 7.523 | 3.745 | 1.865 | Hγ12 0.720  Hγ13 0.621 |  |  |  |
| **Gly^775^** | 7.949 | Qα 3.802 |  |  |  |  |  |
| **Trp^776^** | 7.750 | 4.474 | Hβ2 3.796  Hβ3 3.306 |  | Hδ1 7.076 | Hε1 10.362  Hε3 7.386 | HH2 6.897  Hζ2 7.254  Hζ3 6.854 |
| **Ile^777^** | 7.450 | 3.886 | 1.828 | Qγ1 1.406  Qγ2 1.082 | Qδ1 0.848 |  |  |
