# Supplementary material for: Structural basis of antiviral activity of peptides from MPER of FIV gp36
Source: PLoS One. 2018 Sep 21;13(9):e0204042. doi: 10.1371/journal.pone.0204042 (PMC6150481; doi:10.1371/journal.pone.0204042)
Supplement: S2 Table — 1H chemical shift of C6b in DPC/SDS micelle solution 90:10 M/M. (DOCX) [file pone.0204042.s002.docx]

**S2 Table.** **^1^H chemical shift of C6b.** ^1^H chemical shift of C6b in DPC/SDS micelle solution 90:10 M/M.

| **Residue** | **HN** | **CαH** | **CβH** | **CγH** | **CδH** | **CεH** | **Others** |
| --- | --- | --- | --- | --- | --- | --- | --- |
| **Trp^770^** | 8.407 | 4.263 | Qβ 3.193 |  | Hδ1 6.763 | Hε1 10.454  Hε3 7.397 | Hζ2 7.360 |
| **Glu^771^** | 8.420 | 3.852 | Hβ2 1.817  Hβ3 1.738 | Hγ2 2.087  Hγ3 2.039 |  |  |  |
| **Asp^772^** | 8.022 | 4.464 | Qβ 2.765 |  |  |  |  |
| **Trp^773^** | 7.928 | 4.416 | Hβ2 3.373  Hβ3 3.273 |  | Hδ1 6.972 | Hε1 10.447  Hε3 7.534 | Hζ2 7.255 |
| **Val^774^** | 7.735 | 3.859 | 2.126 | Qγ1 0.944  Qγ2 0.905 |  |  |  |
| **Gly^775^** | 7.784 | Qα 3.801 |  |  |  |  |  |
