# Supplementary material for: Structural basis of antiviral activity of peptides from MPER of FIV gp36
Source: PLoS One. 2018 Sep 21;13(9):e0204042. doi: 10.1371/journal.pone.0204042 (PMC6150481; doi:10.1371/journal.pone.0204042)
Supplement: S3 Table — Molecular surface values calculated as propensities to engage molecular interaction according to specific surface properties. (DOCX) [file pone.0204042.s003.docx]

**S3 Table.** **Molecular surface values.** Molecular surface values calculated as propensities to engage molecular interaction according to specific surface properties.

| Peptide | Hydrophobic surface | Negatively charged surface | Positively charged surface |
| --- | --- | --- | --- |
| **C8** | 288.3 Å^2^ | 210.8 Å^2^ | 137.9 Å^2^ |
| **C6a** | 286.2 Å^2^ | 116 Å^2^ | 106 Å^2^ |
| **C6b** | 220.5 Å^2^ | 177.6 Å^2^ | 135.8 Å^2^ |
