# Supplementary material for: Structural basis of antiviral activity of peptides from MPER of FIV gp36
Source: PLoS One. 2018 Sep 21;13(9):e0204042. doi: 10.1371/journal.pone.0204042 (PMC6150481; doi:10.1371/journal.pone.0204042)
Supplement: S4 Table — Backbone dihedral angles of C6a and C6b in DPC/SDS 90:10 M/M micelle solution. (DOCX) [file pone.0204042.s004.docx]

**Table S4.** Backbone dihedral angles of C6a and C6b in DPC/SDS 90:10 M/M micelle solution.

| **C6a** |  |  |  | **C6b** |  |  |
| --- | --- | --- | --- | --- | --- | --- |
| **Residue** | **Φ(phi)** | **Ψ(psi)** |  | **Residue** | **Φ(phi)** | **Ψ(psi)** |
| Asp^772^ |  |  |  | Trp^770^ |  |  |
| Trp^773^ | -48 | -27 |  | Glu^771^ | -78 | 55.9 |
| Val^774^ | -127 | 12.3 |  | Asp^772^ | -174 | -18 |
| Gly^775^ | -126 | -25 |  | Trp^773^ | -123 | -63 |
| Trp^776^ | -51 | -23 |  | Val^774^ | -80 | -20 |
| Ile^777^ |  |  |  | Gly^775^ |  |  |
