# Supplementary material for: Structural basis of antiviral activity of peptides from MPER of FIV gp36
Source: PLoS One. 2018 Sep 21;13(9):e0204042. doi: 10.1371/journal.pone.0204042 (PMC6150481; doi:10.1371/journal.pone.0204042)

**S1 Fig.** **Sequential and medium range connectivities of C6a and C6b.** Sequential and medium range connectivities collected in NOESY spectra of C6a and C6b in DPC/SDS 90:10 M/M.


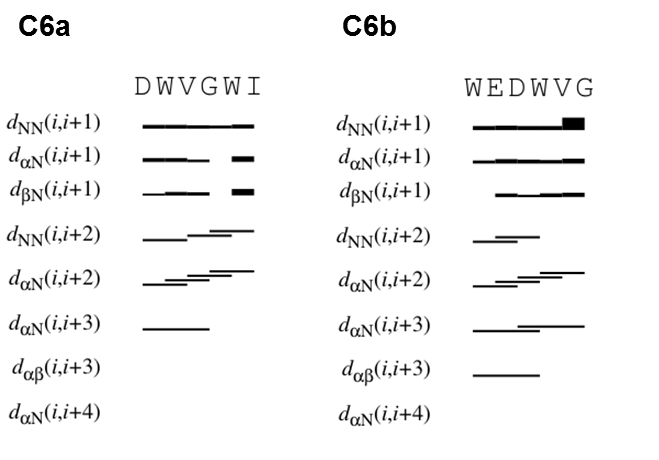

Supplement: S1 Fig — Sequential and medium range connectivities collected in NOESY spectra of C6a and C6b in DPC/SDS 90:10 M/M. (DOCX) [file pone.0204042.s005.docx]
