# Supplementary material for: Structural basis of antiviral activity of peptides from MPER of FIV gp36
Source: PLoS One. 2018 Sep 21;13(9):e0204042. doi: 10.1371/journal.pone.0204042 (PMC6150481; doi:10.1371/journal.pone.0204042)

**S2 Fig.** **Low field region of the 600 MHz 1D proton spectra of C6a (a) and C6b (b) peptides** **in presence of spin labels.** Low field region of the 600 MHz 1D proton spectra of C6a (a) and C6b (b) peptides recorded in DPC/SDS 90:10 M/M micelles solutions at 300 K in absence (blue) and in presence of 5-DSA and 16-DSA (red) at a concentration of one spin label *per* micelle.


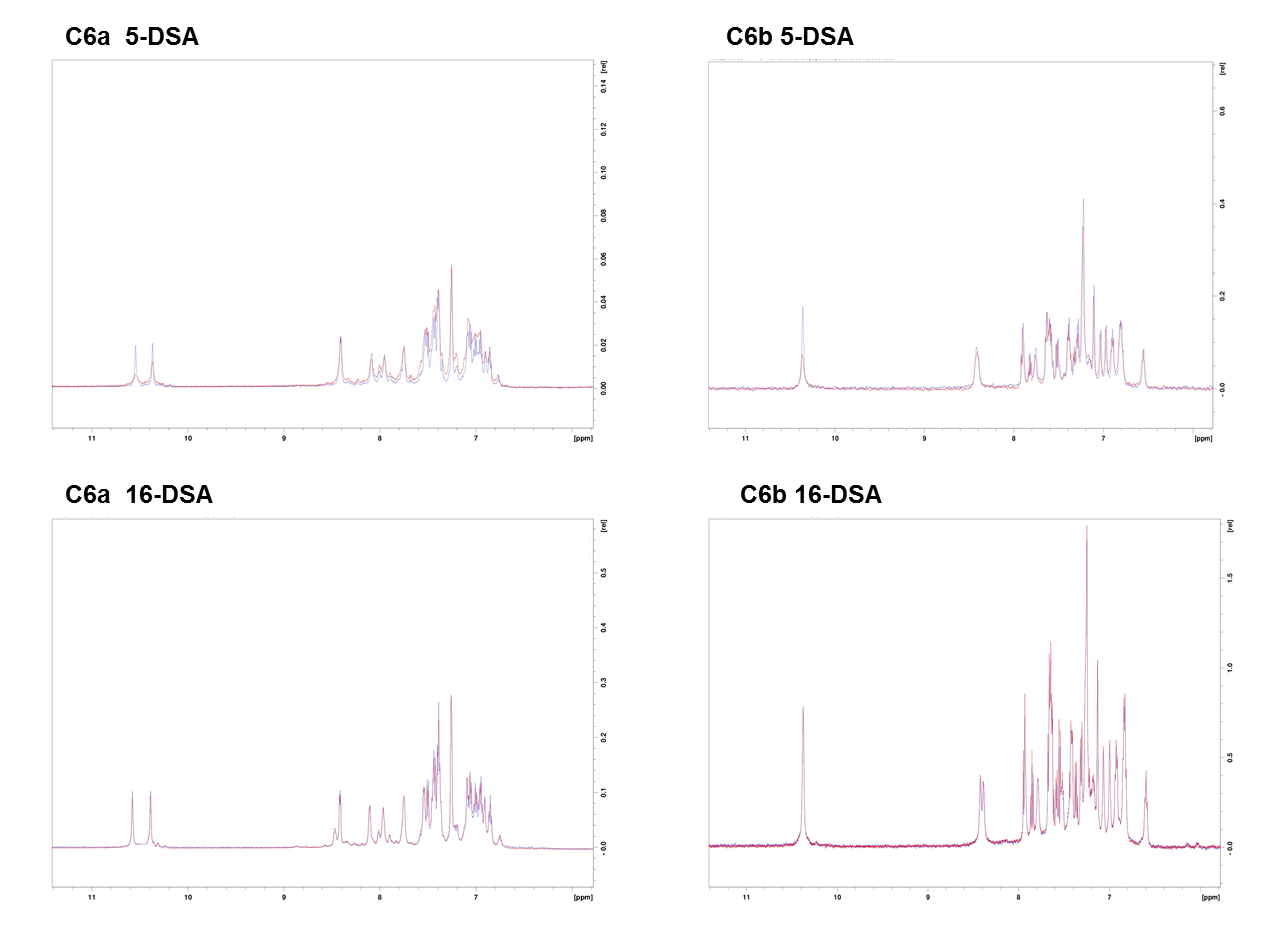

Supplement: S2 Fig — Low field region of the 600 MHz 1D proton spectra of C6a (a) and C6b (b) peptides in presence of spin labels. Low field region of the 600 MHz 1D proton spectra of C6a (a) and C6b (b) peptides recorded in DPC/SDS 90:10 M/M micelles solutions at 300 K in absence (blue) and in presence of 5-DSA and 16-DSA (red) at a concentration of one spin label per micelle. (DOCX) [file pone.0204042.s006.docx]
